# Frequency of Exposure of Nephrotoxic Drugs and Drug-Induced Acute Kidney Injury in Pediatric Intensive Care Unit: A Retrospective Review From a Tertiary Care Centre in Pakistan

**DOI:** 10.7759/cureus.12183

**Published:** 2020-12-20

**Authors:** Rahim Ahmed, Muhammad Shahzad, Anum Umer, Asim Azim, Muhammad Tariq Jamil, Anwar Haque

**Affiliations:** 1 Pediatric Intensive Care Unit, The Indus Hospital, Karachi, PAK; 2 Pediatrics, The Indus Hospital, Karachi, PAK; 3 Pediatrics, Liaquat National Hospital & Medical College, Karachi, PAK

**Keywords:** acute kidney injury, causes, nephrotoxic medications, complications

## Abstract

Introduction

Acute kidney injury (AKI) is one of the most common problems seen in the pediatric intensive care unit (PICU), with an overall 27% incidence. Besides many other factors, nephrotoxic medications (Nephrotoxins; Ntx) are also responsible for a large proportion of potentially avoidable pediatric AKI, directly accounting for 16% of AKI events.

Objective

To assess potential associations between nephrotoxic drugs and the risk of developing AKI in children admitted in PICU.

Material and methods

This is a retrospective cross-sectional study. Children (aged 1 month - 18 years) admitted to the PICU, with a length of stay >24 hours, were included. AKI was defined as according to KDIGO (Kidney Disease Improving Global Outcomes) criteria. Mild AKI was defined as a rise in creatinine value of 0.3 mg/dl from presenting value at a 24-hour interval. Patients were grouped according to the presence or absence of AKI. All medications administered in the ICU were assessed for nephrotoxicity through a review of adverse reactions mentioned in the Pediatric Dosage Handbook, along with consultation with a clinical pharmacist.

Results

Among 752 patients, the mean age was 4.8 years ± 4.37. There were 57.3% male and 42.7% female children. Among the exposed children, 37.4% received one drug, 32.4% received two drugs and 12.1% had high nephrotoxin exposure. The most commonly used drug was vancomycin (16.8%), as a single Ntx; vancomycin/colistin (12.9%), in dual nephrotoxic combination; and vancomycin/colistin/amphotericin (2.9%) in highly exposed children (i.e., with equal or more than three). Overall, the incidence of AKI was 14.9%.

Conclusion

Nephrotoxins are potentially avoidable risk factors in critically ill children. Whenever a combination of medications is required, it’s advisable to review all medications for better protection of kidneys and preventing of acute kidney injury.

## Introduction

Acute kidney injury (AKI) is one of the most common problems seen in the pediatric intensive care unit (PICU), with an overall 27% incidence and has been found a low prognostic factor with increase mortality and length of stay [1]. Besides, children in the PICU may be predisposed to developing AKI based on their underlying medical conditions such as sepsis or treatments before admission to the ICU. Conversely, hemodynamic abnormalities, medical conditions, and therapies like drugs, mechanical ventilation may also predispose them to the risk of developing AKI [2-3]. Mechanical ventilation has been associated as an independent risk factor in the progression towards AKI [4].

More recently, medication-associated nephrotoxicity and the concept of nephrotoxic burden have come to attention. Nephrotoxic medications (Nephrotoxins; Ntx) may lead to AKI, as 16% of AKI events in one study [5]. The predisposing factors associated with Ntx are age, underlying disease, pharmacogenetics of Ntx, dosage, and concomitant medications [6]. Vancomycin has been found in 7% of patients leading to AKI [7].

The aim of our study is to assess potential associations between nephrotoxic drugs and the risk of developing AKI in children admitted to PICU. Therefore, we aimed to find the incidence of AKI after exposure to Ntx in critically ill children of a lower-middle-income country.

## Materials and methods

This is a retrospective cross-sectional study with a chart review from January 2017 till January 2020, conducted in the PICU of a tertiary-care hospital in Pakistan after approval from the ethical review committee. All children (aged one month - 18 years) admitted to the PICU, with a length of stay >24 hours were included. Children with known chronic illness taking any Nephrotoxins, creatinine with more than 1 mg/dl at presentation, expire in 24 hours of admission, and end-stage renal disease was excluded from the study.

Demographic and clinical data were collected from patients’ medical records. Renal function was defined as the first serum creatinine measurement within 24 hours of ICU admission. Serum creatinine was recorded at presentation, 24 hours, and 48 hours interval. KDIGO (Kidney Disease Improving Global Outcomes) criteria were used to define the AKI with rise in creatinine value of 0.3 mg/dl within 24 hours as mild AKI. Patients were labeled as AKI present or not.

All medications administered in the ICU were assessed for nephrotoxicity through a review of adverse reactions mentioned in the Pediatric Dosage Handbook, along with consultation with a clinical pharmacist [8]. Nephrotoxins were defined as the Nephrotoxic Injury Negated by Just-in-time Action (NINJA) collaborative. Medication dispensing and administration data were extracted from the electronic medication record (EMR). Nephrotoxins were counted individually, and high exposure was defined as receipt of ≥3 Ntx concurrently per the NINJA collaborative [9].

The primary outcome was the occurrence of AKI with the degree of nephrotoxic exposure.

Age was categorized as infants (1-12 months), toddlers (1-4 years), children (5-11 years), and adolescents (>12-18 years). Continuous data were summarized with descriptive statistics (mean ± standard deviation). While statistical differences between the mean values were compared using Student's t-test. Categorical variables were summarized using frequency and proportion. Statistical analyses were performed using SPSS version 23.0 (IBM Corp., Armonk, NY). A p-value < 0.05 was considered statistically significant.

## Results

We collected the data of 871 children admitted in PICU and excluded 88 patients who presented with creatinine more than 1 mg/dl at presentation. We also excluded 31 patients who expired within 24 hours of admission. Among 752 patients, the mean age was 4.8 years ± 4.37. There were 57.3% male and 42.7% female children. A total of 451 patients (60%) were admitted from the emergency room. While 616 children (81.9%) received at least one nephrotoxic drug, 136 children (18.1%) received no nephrotoxic drug from the list shown below (Table 1). On the basis of KDIGO criteria, AKI was present in 112 children (14.9%).

**Table 1:** Demographic and clinical variables

Demographic and clinical variables	n = 752
Age:	4.8 years ± 4.37
Gender; Male: Female	(431:321) 57.3%:42.7%
Creatinine at presentation (mg/dl)	0.42 (0.11-0.99)
Creatinine at 48 presentation	0.53 (0.10-3.10)
Nephrotoxic drugs; Yes: No	616 (81.9%): 136 (18.1%)
Incidence of AKI; Yes: no	112 (14.9%):640 (85.1%)
Mechanical ventilation; Yes: AKI-No: AKI	360:63 (17.5%)-392:49 (12.5%)

We counted 14 drugs that were used in 60 various combinations in our settings (Table 2).

**Table 2:** Commonly used nephrotoxins

Common nephrotoxic drugs used in our settings	Other nephrotoxic drugs
Acyclovir, Amphotericin, Amikacin, Colistin, Ceftazidime, Captopril, Ketorolac, Levetiracetam, Meropenem, Mannitol, Phenytoin, Topiramate, Tazobactam/piperacillin, Vancomycin	Enalapril, Tobramycin, Ticarcillin/clavulanic acid, Gentamicin, Ifosfamide, Ibuprofen, Aspirin

Single commonly used drugs were vancomycin (16.8%), tazobactam/piperacillin (12.9%), levetiracetam and colistin (1.2%). In dual nephrotoxic combination, vancomycin/colistin (12.9%), vancomycin/levetiracetam (5/9%) and vancomycin/tazobactam/piperacillin (3.3%) were used. In highly exposed children, vancomycin/colistin/amphotericin (2.9%), vancomycin/levetiracetam/phenytoin (2.9%), vancomycin/colistin/levetiracetam (1.7%) were used (Table 3).

**Table 3:** Single and cumulative nephrotoxins frequency and incidence of acute kidney injury (AKI)

Number of drugs	Common drugs	Total usage	Incidence of AKI	P-value
One drug use	Vancomycin	126 (16.8%)	24 (19%)	0.07
	Tazobactem/piperacillin	97 (12.9%)	09 (9.3%)	
	Levetiracetam	18 (2.4%)	02 (11.1%)	
	Colistimethate	09 (1.2%)	01 (11.1%)	
Two drug use	Vancomycin Colistimethate	97 (12.9%)	22 (22.6%)	0.03
	Vancomycin, Tazobactem/piperacillin	25 (3.3%)	5 (20%)	
	Vancomycin Acyclovir	4 (0.5%)	1 (25%)	
Three or more drug use	Vancomycin, Colistimethate, Amphotericin	22 (2.9%)	4 (18.1%)	0.09
	Vancomycin, Levetiracetam, Phenytoin	22 (2.9%)	2 (9%)	
	Vancomycin, Colistimethate, levetericetam	13 (1.7%)	4 (30.7%)	

We found the incidence of AKI around 14.9% in all enlisted patients. In AKI patients, 22 (19.6%) did not receive the nephrotoxic drug while 41 (36.6%) received one drug, 39 (34.8%) two, and 10 (8.9%) three or more drugs.

## Discussion

Usage of medications with nephrotoxicity is highly prevalent in intensive care units. Additionally, 44.5% of patients were exposed to two or more nephrotoxic medications. Among these patients, we found that 12.1% received higher nephrotoxins than other studies, in which 3.3% of patients received nephrotoxins [10-11]. Our findings suggest that children in the PICU are at significantly higher risk for nephrotoxin exposure. Now, nephrotoxic-medication exposure is becoming more prevalent as a primary cause of AKI, comprising approximately 16% of all pediatric inpatient causes of AKI [12].

The incidence of AKI was 14.9% in the exposed group. Although that was contrary to other studies in which the incidence was relatively high, like Uber et al. [13]. This study was conducted in post-cardiac surgical patients in which the incidence of AKI is more than the general population because of multiple risk factors.

Vancomycin was found to be the most frequently used nephrotoxic medication in our PICU. Totapally et al. have reported a high blood urea nitrogen (BUN): Creatinine ratio with exposure to nephrotoxic drugs (odds ratio 2.23, CI 1.27-3.93). Among these, vancomycin was associated with AKI [14].

We did find an independent association between single exposure to the drug and the development of AKI. As drugs were used collectively, there was an increased incidence of AKI, although the statistical association was less significant. It may be explained by the involvement of co-factors like mechanical ventilation and inotropes that were used with the increased severity of critical illness. As in one study conducted by Uber et al., ketorolac was the most common drug used, and the incidence of AKI occurred more commonly in those with more than three drugs, but it was not significant because of confounders [13].

Critically ill patients have constantly fluctuating pharmacokinetics because of changing serum creatinine and alterations in fluid status, which makes medication dosing tricky and adds to the risk of AKI [15]. Meanwhile, a high enough medication dose to ensure adequate concentration at the site of infection and addressing the bacterial resistance needs to focus while avoiding medication toxicity with renal effects [16].

As mechanical ventilation has been considered an independent risk factor for AKI [17], we calculated the incidence of AKI in ventilated and non-ventilated patients related to the use of nephrotoxic drugs. Incidence of AKI was more in ventilated patients, but non-ventilated patients also had a significant occurrence of AKI (p-value 0.05).

Health outcomes of pediatric patients may be improved by identifying patients at risk for medication-associated AKI. Monitoring strategies and medication selection may need to be altered to prevent poor outcomes or minimize the risk caused by nephrotoxic medications.

We took the data of the last two years, and a large number of patients gave us a broad way to find a significant correlation. In many other studies, the group of patients was small [5,13].

Acute dialysis quality initiative (ADQI) work group has started to develop a consensus for the design of automated AKI detection systems to produce real-time AKI alerts using electronic networks [18]. We also recommend making it available locally in addition to notifying the combination of nephrotoxic drugs.

We have a few limitations to our study. It was a retrospective study conducted on admitted patients in two years. While our research finds a weak statistical association between exposure to Ntx and AKI, but this entity needs more extensive multi-center trials. Regardless, our data suggest that nephrotoxin use remains a modifiable AKI risk factor.

## Conclusions

Utilization and combinations of nephrotoxins in PICU is quite high. Some combinations are more toxic than others. Also, Ntx have been found to correlate with the occurrence of AKI. Whenever a combination of medications is required, it is advisable to review all medications for better protection of kidneys.

Among children who were on inotropic support or on mechanical ventilation, we have found a more incidence of AKI.
